# Unfortunate

**Published:** 1881-10-20

**Authors:** 


*Unfortunate.*—The bill passed by the late Georgia Legislature, designed to facilitate and legalize dissections, was vetoed by Governor Colquitt. So it is that the law-making power continues to exercise the inconsistency of holding us liable to prosecution for malpractice for ignorance of anatomy, while imposing obstacles to the only means of obtaining the required information.

